# The effectiveness of lamotrigine for persistent depressive disorder: A case report

**DOI:** 10.1002/npr2.12228

**Published:** 2022-01-05

**Authors:** Yusuke Matsuzaka, Kayoko Urashima, Shintaro Sakai, Yoshiro Morimoto, Shinji Kanegae, Hirohisa Kinoshita, Akira Imamura, Hiroki Ozawa

**Affiliations:** ^1^ Department of Neuropsychiatry Nagasaki University Hospital Nagasaki Japan; ^2^ Department of Neuropsychiatry Nagasaki University Graduate School of Biomedical Sciences Nagasaki Japan; ^3^ Health Center Nagasaki University Nagasaki Japan; ^4^ Child and Adolescent Psychiatry Community Partnership Unit Nagasaki University Hospital Nagasaki Japan

**Keywords:** bipolar and related disorders, lamotrigine, persistent depressive disorder (PDD)

## Abstract

**Aim:**

Persistent depressive disorder (PDD) was first introduced in the Diagnostic and Statistical Manual of Mental Disorders 5th edition (DSM‐5), which encompasses numerous different conditions, including dysthymia, recurrent major depressive disorder, double depression, and chronic major depression. SSRIs are the first‐line drugs for treatment of PDD; however, not all patients respond to SSRI treatment.

**Case presentation:**

We describe a woman who was diagnosed with PDD. At the age of 38, the patient presented with anxiety, reduced energy, marked tiredness, and sleep disturbances. She was prescribed with three antidepressants (paroxetine, duloxetine, and mirtazapine), which were not effective in relieving her symptoms. She was also prescribed bromazepam, which was also not effective. Subsequently, she was switched to lamotrigine, which resulted in a marked improvement in symptoms. The antidepressants and bromazepam were gradually tapered and discontinued.

**Conclusion:**

This case demonstrates that lamotrigine may be effective for treating patients with antidepressant resistant PDD and suggests that it may be a promising alternative to combination therapy of antidepressants and benzodiazepines in the treatment of PDD.

## Abstract 2

The patient's depressive and anxiety symptoms remitted and relapsed repeatedly during taking combination therapy. After switching to lamotrigine monotherapy, the symptoms have improved and continued remission for a long time.

## Graphical abstract

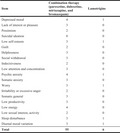

## 1 INTRODUCTION

Persistent depressive disorder (PDD) was first introduced in the Diagnostic and Statistical Manual of Mental Disorders 5th edition (DSM‐5), which encompasses numerous different conditions, including dysthymia (DST), recurrent major depressive disorder, double depression, and chronic major depression. Since its inception in the DSM‐III, DST has been widely criticized for its significant heterogeneity of the diagnosis, and these criticisms have continued with PDD, its latest classification. Several reports have examined the efficacy and acceptability of selective serotonin reuptake inhibitors (SSRIs) and tricyclic antidepressants in the treatment of PDD^1^ and have provided evidence for the efficacy of antidepressants in the treatment of chronic depression. However, reports have shown that up to two‐thirds of adult patients do not achieve remission with SSRI treatment. Moreover, there is limited evidence identifying reliable predictors (eg, demographic, clinical, or genetic characteristics) of individual response.^2^, ^3^ Therefore, effective treatment of patients with PDD who do not respond to SSRIs remains elusive.

Here, we report a patient who was diagnosed with PDD who showed insufficient improvement with a combination therapy of antidepressants but achieved remission with lamotrigine monotherapy.

## 2 CASE REPORT

The patient was a woman in her 50’s. Her mother, grandmother, and aunt were diagnosed with major depressive disorder and committed suicide. She worked in a hospital as a nurse. At the age of 38 years, she began experiencing fatigue and wobble. She visited a general medicine clinic, but no abnormalities were detected. She presented with symptoms of depression (eg, depressed mood, reduced energy, pessimism, and sleep disturbances). She did not present with hypomanic or manic symptoms, such as mood elevation. At the psychiatry clinic, she was diagnosed with major depressive disorder and was started on paroxetine. However, her symptoms remained unstable, and she remitted and relapsed repeatedly. Her diagnosis was revised from major depressive disorder to PDD, and duloxetine and mirtazapine were added to her treatment when she was aged 45 and 46 years, respectively. She was prescribed maximum doses of paroxetine, duloxetine, and mirtazapine; however, her symptoms did not improve. Furthermore, she was prescribed the maximum dose of bromazepam for anxiety, but her anxiety symptoms persisted. Although the patient had treatment resistance to antidepressants, she did not experience severe depressive symptoms (such as suicidal ideation) or psychotic symptoms (such as delusions); thus, augmentation therapy with antipsychotics and electroconvulsive therapy were considered unsuitable. Subsequently, she was treated with lamotrigine and her symptoms of depressions stabilized. The three antidepressants were gradually tapered and discontinued. Her anxiety also improved dramatically, and bromazepam was gradually tapered and discontinued. Remission was maintained for over 2 years, and she was reemployed as a care insurance investigator.

Detailed information of the patient's symptoms (measured using the Cornell Dysthymia Rating Scale^4^) is summarized in Table 1.

**TABLE 1:** Clinical information of the patients according to the Cornell Dysthymia Rating Scale

Item	Combination therapy (paroxetine, duloxetine, mirtazapine, and bromazepam)	Lamotrigine
Depressed mood	4	1
Lack of interest or pleasure	3	0
Pessimism	2	0
Suicidal ideation	0	0
Low self‐esteem	3	1
Guilt	2	0
Helplessness	3	0
Social withdrawal	3	0
Indecisiveness	2	0
Low attention and concentration	3	0
Psychic anxiety	4	1
Somatic anxiety	3	0
Worry	3	1
Irritability or excessive anger	2	0
Somatic general	3	0
Low productivity	3	0
Low energy	4	0
Low sexual interest, activity	2	0
Sleep disturbance	3	1
Diurnal mood variation	3	1
Total	55	6

Note

After switching to lamotrigine monotherapy, the symptoms have improved and continued remission for a long time.

## 3 DISCUSSION

Lamotrigine has not showed efficacy in the treatment of unipolar depression.^5^, ^6^ This case provided the new findings that lamotrigine improved unipolar depression resistant to antidepressants and also improved anxiety symptoms being free from benzodiazepines.

Our patient presented with two contradictory aspects: (a) She met criteria for PDD according to the DSM‐5 but did not meet criteria for bipolar disorder; and (b) although she did not show improvement with antidepressant treatment, remission was achieved following therapy with a mood stabilizer, lamotrigine. There are several guidelines that recommend mood stabilizers for treating patients with depressive disorder based on the recognition of bipolar disorder as a broad‐spectrum disorder that encompasses depressive disorder.^7^, ^8^, ^9^ Moreover, PDD is thought to be a predictive factor for bipolar disorder, alongside family history of bipolar disorder and cyclothymic temperament.^10^ Therefore, the concepts of bipolar disorder and PDD need to be considered carefully.

There has been limited discussion around the use of mood stabilizers for the treatment of PDD. Most mood stabilizers are effective for managing manic states, but they are not effective for resolving depressive states. However, lamotrigine is effective for the treatment of bipolar depression and has a low risk of manic switch.^11^ Furthermore, lamotrigine has a therapeutic effect on depressive cognition and psychomotor retardation in patients with bipolar depression.^12^ Therefore, the use of lamotrigine in the treatment of PDD may be useful in regard to efficacy and safety. In animal models, the blocking of sodium channels was related to improving depressive symptom.^13^

Interestingly, our patient presented with depression‐related anxiety, which also improved with lamotrigine. Anxiety frequently coexists with depression, and the addition of benzodiazepines to antidepressant treatment is common practice in treatments for major depression. Reports have shown that combination therapy with antidepressants and benzodiazepines is more effective than antidepressant monotherapy for improving the severity, treatment response, and remission in the early stages of depression.^14^ However, in our case, combination therapy of antidepressants and benzodiazepines was not effective. Moreover, animal models have revealed that lamotrigine has an anxiolytic‐like pharmacokinetic profile that may be related to sodium channels.^15^ Taken together, we suggest that lamotrigine is a promising alternative to benzodiazepines in the treatment of PDD.

The plasma concentration of lamotrigine has not been measured in this case. Therapeutic response to lamotrigine occurs when its plasma concentration is higher than 12.7 μmol/L.^16^ This response was derived in 75‐100 mg dosage, so our patient might have sufficient therapeutic effect of lamotrigine with 200 mg.

In summary, our case demonstrated that lamotrigine may be effective in patients with PDD who are resistant to antidepressants. Moreover, lamotrigine may be a promising alternative to combination therapy of antidepressants and benzodiazepines in the treatment of PDD. Fully understanding the homogeneity and heterogeneity of bipolar disorder and PDD requires further clinical and biological investigations. Nevertheless, we believe that our case provides an opportunity to further our understanding of these disorders.

## CONFLICT OF INTEREST

None.

## AUTHOR CONTRIBUTIONS

Yu. M. treated the patient and drafted the manuscript. Yo. M. critically reviewed the draft and revised it. All authors made substantial contributions, drafted the manuscript, and approved the final manuscript.

## INFORMED CONSENT

Written consent from the patient was obtained.

## Data Availability

Data sharing is not applicable to this article as no datasets were generated or analyzed during the current study.
